# The mechanical role of the metatarsophalangeal joint in human jumping

**DOI:** 10.1371/journal.pone.0268634

**Published:** 2022-05-20

**Authors:** Junichiro Yamauchi, Keiji Koyama

**Affiliations:** 1 Tokyo Metropolitan University, Tokyo, Japan; 2 Toin University of Yokohama, Kanagawa, Japan; Mugla Sitki Kocman Universitesi, TURKEY

## Abstract

This study investigated the mechanical role of metatarsophalangeal (MTP) joints in human jumping. Eighteen healthy young men performed three types of single-leg jumps (SJ: squat jump; CMJ: countermovement jump; HJ: standing horizontal jump) on a force plate under barefoot (BARE) and forefoot immobilisation (FFIM) conditions. For FFIM, the forefoot was immobilised around the MTP joints of the dominant leg by a custom-made splint. Force-time components and the centre of pressure (COP) trajectory were measured from the ground reaction force (GRF) in the take-off phase of jumping. The vertical jump heights calculated from the net vertical impulse were lower under FFIM than under BARE during the CMJ (p < 0.05). The HJ distance under FFIM was significantly shorter than that under BARE (p < 0.01). The relative net vertical impulse was lower under FFIM than under BARE during the CMJ (p < 0.05). During the HJ, all the horizontal GRF variables were significantly lower under FFIM than under BARE (p < 0.01), but none of the vertical GRF variables differed between the two conditions. The horizontal relative GRF in the 90–95% of the final take-off phase during the HJ was significantly lower under FFIM than under BARE (p < 0.01). Under FFIM, the COP range in the antero-posterior direction in the take-off phase of the HJ decreased (p < 0.05), whereas its range in the anterior direction for the SJ and CMJ increased (p < 0.05). The results of this study indicate that MTP joint motion can play an important role in regulating force-generating capacities of toe flexor muscles in the take-off phase of human jumping, especially in the horizontal direction of horizontal jumping.

## Introduction

Humans could potentiate the force-generating capacity of the toe flexors at the metatarsophalangeal (MTP) joints in concert with the foot arch modification during the push-off phase in bipedal locomotion. Throughout the evolutional process, the human foot has adapted to be a large heel bone, short toes, the torsion of the metatarsal heads and the adduction of the hallux [1]. These features of the foot made it possible for human to stand and walk with a plantigrade position, and to run and jump with a digitigrade position in bipeds. The toe flexor muscles generate force during the ground contact of human biped locomotion as the MTP joints are dorsiflexed [2]. Dorsiflexion of the toes induces the winding of the plantar fascia around the metatarsal heads [3]. This windlass effect occurs when the foot is ready to lift off the ground during the gait cycle [4] and the take-off phase of jumping [5]. The toe flexor muscles are able to potentiate force generation with the windlass effect in the human biped locomotion [6]. Also, the shorter toes reduce MTP joint moments and require less force production of toe flexor muscles during running [7]. Consequently, these indicate that the mechanical contribution of the MTP joints could reflect the force components on the ground during human biped performance.

Considering that MTP joint motion is a key mechanism for regulating the force generation of the toe flexor muscle and tendon complex during human biped locomotion, it is speculated that the force generation of toe flexor muscles at the MTP joints could help to propel the body from the ground, especially in the final take-off trajectory of jumping. The toe flexor muscles generate internal moments at the MTP joints and lift the heel off the ground during jumping [5,8]. Hence, the maximum toe flexor strength was significantly correlated with dynamic lower-limb physical performance, such as sprinting and horizontal jumping in children [9] and adolescents [10], and vertical jump performance in adults [11]. Horizontal jump performance is improved after strength training of the toe flexor muscles [5,12]. Also, toe flexor muscles could help reinforcing the structure of a medial longitudinal arch and absorbing impact against an external load [13]. A vertical force on the foot enhances the contact force on the ground by changing the form of the foot arch during dynamic upright locomotion [14]. Indeed, a force generating capacity of toe flexor muscles increased with decreasing foot arch height under the loading condition [15]. It should be aware that force generation at the MTP joint is not only determined by the toe flexor muscle itself but also by the foot arch dynamics.

This mechanical function of the MTP joint could be altered by increasing the bending stiffnesses of footwear during running and jumping [16–18]. A systematic review highlights that forefoot bending stiffness of footwear is considered to be one of the external tools to optimize physical performance artificially [19]; however, the motion of footwear is different from the actual motion of the foot [20]. In the clinical situation, reduced MTP dorsal flexion mobility of rheumatoid arthritis patients is associated with impaired walking parameters, such as walking velocity and stride length [21]. Another study shows that elastic strapping around the MTP joints increases vertical jump performance with increasing forefoot bending stiffnesses of the foot; however, the high pressure of MTP joint strapping is intended to restrict free joint motion and not to help improving jump performance to a large extent [22]. Therefore, it would be interesting to investigate how MTP joint motion contributes to the force components on the ground during jumping when its motion is experimentally restricted by the immobilisation of the forefoot; however, previous studies have not articulated how MTP joint motion aids human jumping practically, nor have they demonstrated whether the immobilised MTP joint motion affects the force components on the ground during jumping. Therefore, the purpose of this study was to investigate the mechanical role of the MTP joint in vertical and horizontal jump performance. It was hypothesized that the immobilisation of the forefoot would prevent the stretching and shortening of the muscle and tendon complex of the foot at the MTP joints and impair the function of the foot arch dynamics, thus reducing the force on the ground in the take-off phase of jumping and decreasing jump performance. The results of this study could provide insight as to the mechanism by which the MTP joint motion influences jump performance.

## Methods

### Subjects

Eighteen healthy young men (age: 20.6 ± 0.9 yrs; height: 1.70 ± 0.07 m; body mass: 64.9 ± 9.5 kg; mean ± SD) volunteered to participate. After a preliminary screening, which included a medical history and fitness profile, participants were included if they had no history of serious foot or leg injury or surgery, were taking no medications, had no foot deformities, had no experience of metatarsal taping, and had no experience of any jump exercise training within the past 3 months. The required sample size was estimated from a previous study showing that rheumatoid arthritis patients reduced MTP dorsal flexion mobility and slowed walking velocity [21]. The sample size was calculated using differences in walking velocity of rheumatoid (1.21 ± 0.17 m/s) and non-rheumatoid (1.34 ± 0.07 m/s) patients. A significance level of less than 0.05 (Zα/2 (0.025) = 1.96) and 95% power of the test were used for the calculations. According to this calculation, the minimum required sample size was 18. All of the participants were informed about the experimental procedure and the purpose of the study prior to the study onset. Written consent was obtained from all participants. The methods and all procedures used in these experiments were in accordance with the current local guidelines and the Declaration of Helsinki and were approved by the local Ethical Committee for Human Experiments.

### Experimental procedure

The participants were randomly assigned to perform jumps under barefoot (BARE) or forefoot immobilisation (FFIM) conditions, and then they were assigned to perform three types of single leg jumps in the following order: squat jump (SJ), countermovement jump (CMJ) and standing horizontal jump (HJ). Before the maximum measurement, the participants participated in a familiarization session at a submaximum level. For the measurement of the maximum performance, three trials of each jumping condition were performed with 1 minute of the recovery periods among the trials and 3 minutes of the recovery periods between two conditions. Force-time variables and the centre of pressure (COP) trajectory were measured from the ground reaction force (GRF) on a force plate (Kistler, Winterthur) during jumping. A force plate was mounted flush with a carpet floor covering. The dominant leg was selected for all single leg jumps. Before the measurement of the jumps, leg dominance was determined after performing three trials of three functional tests: i) the leg used to perform the step-up on the box, ii) the leg stepped out when participants were pushed from the behind, iii) the leg used to kick the ball [23]. The leg that was dominant in two out of three tests was considered the dominant leg for this study.

### Forefoot immobilisation (FFIM)

**Fig 1:**
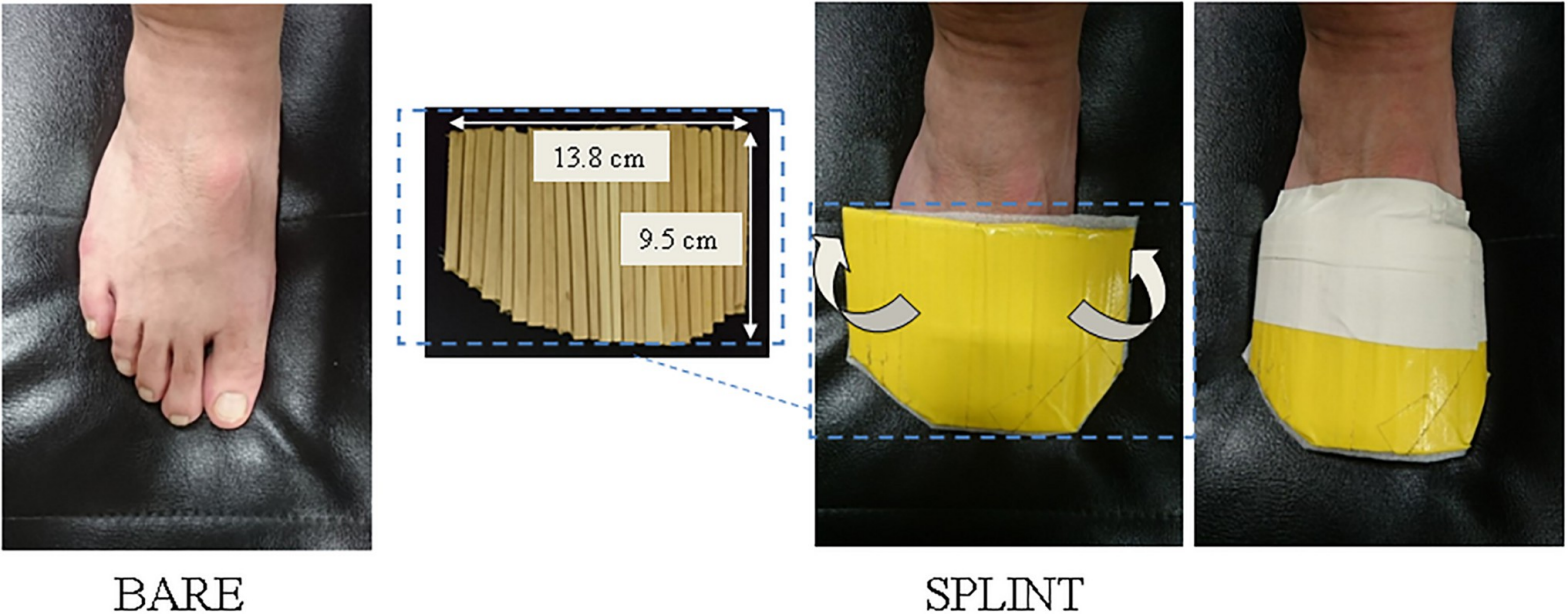
Forefoot immobilisation (FFIM) with a splint. Several pieces of wood sticks were cut and adjusted to the length of the forefoot, and these pieces were jointed parallel with vinyl tape so that this splint could be bent in a small range at the sagittal plane of the foot. The size of the splint was approximately 9.5 cm long, 13.8 cm wide and 48.0 g. The splint was fixed on the dorsal side of the dominant forefoot by taping. White arrows show the direction of overlap around the dorsal side of the forefoot.

The forefoot of the dominant leg was immobilised with a custom-made splint (Fig 1). The splint was made from wood sticks (0.4~0.6 cm wide of light wood). Several pieces of wood sticks were cut and adjusted to the length of the forefoot, and these pieces were jointed parallel with vinyl tape so that this splint could be bent in a small range at the sagittal plane of the foot. The size of the splint was approximately 9.5 cm long, 13.8 cm wide and 48.0 g. The splint was fit comfortably on the dorsal side of the dominant forefoot and secured around the forefoot with standard athletic tape (38.0 mm・13.7 m, Nitreat CB-V, Nitto Medical Corporation, Osaka). This split was to rigidify all forefoot joints, ensuring no active MTP joint motion.

### Evaluation of jump performance

#### Jump protocol

Three types of jumping (SJ, CMJ, HJ) using a single leg without arm swing were performed on a force plate. The details of the methodology for the vertical jump measurements have been described elsewhere [24]. The CMJ was performed by rapidly moving downward, immediately followed by an explosive upward movement, whereas the SJ was performed from an initial static position with 90 degrees of knee flexion. For both the SJ and CMJ, the participants kept their torso in an upright position to emphasize the use of the leg extensor muscles and attempted to jump as high as possible [25]. The HJ was performed as far as possible in the horizontal direction, and the distance between the start line at the toe of the foot and the landing point at the heel of the foot on the floor was measured. For all jumps, the participants placed their arms akimbo throughout the entire jump. After they performed a number of submaximum jumps, they repeated three maximum jumps with at least 3 minutes of the recovery time between bouts. The maximum values of the three measurements were used for further analysis.

### Data analysis

#### Force-time components

**Fig 2:**
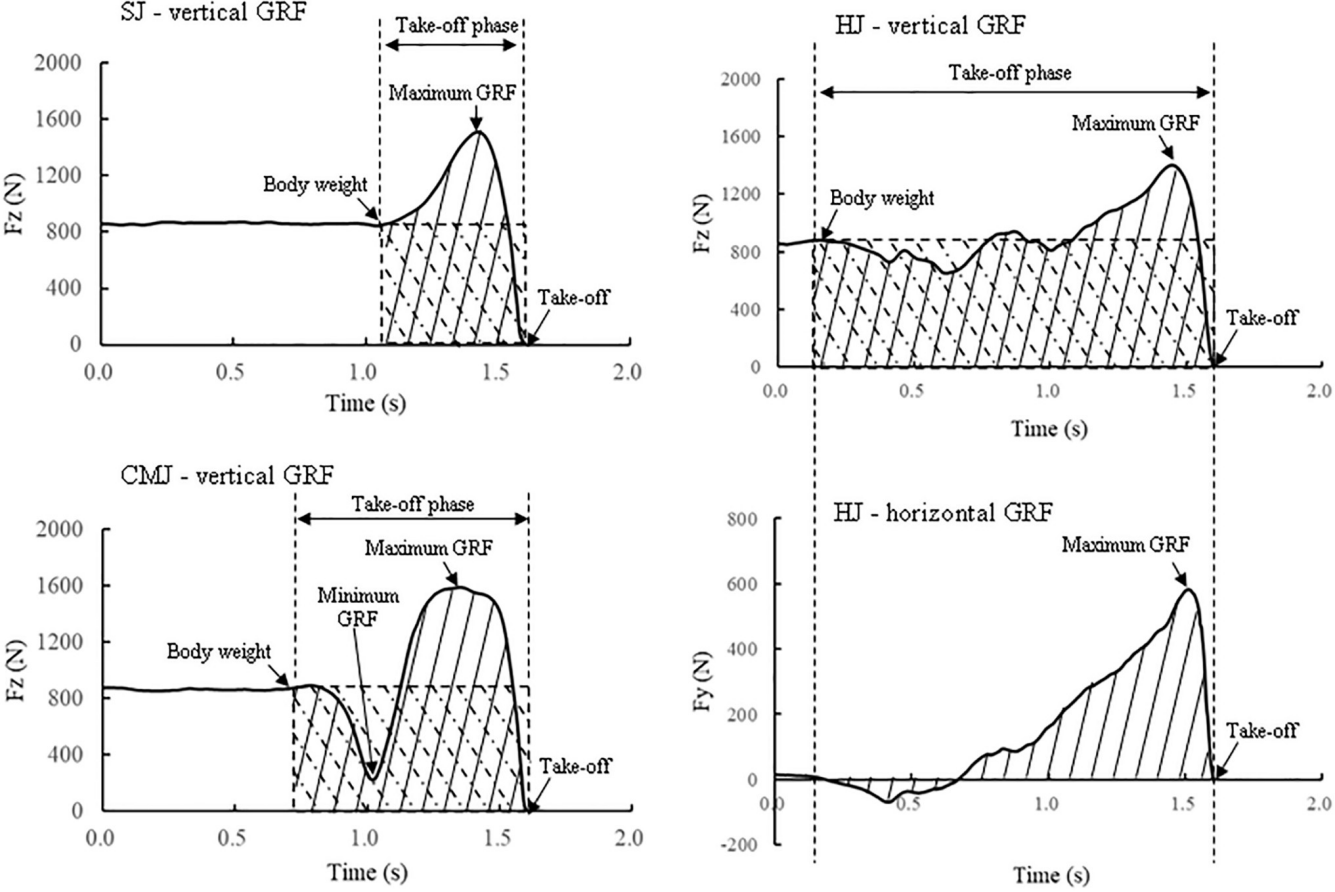
Typical raw data of ground reaction force during the squat jump (SJ), countermovement jump (CMJ) and horizontal jump (HJ) under barefoot conditions. Take-off phase: The range from the onset of the vertical GRF change to the take-off during the ground contact of the SJ, CMJ and HJ; Fz_max: Maximum vertical ground reaction force; Fz_mini: Minimum vertical ground reaction force; Fy_max: Maximum horizontal ground reaction force; VI (grey area): Net vertical impulse; HI (grey area): Net horizontal impulse.

The vertical (Fz) and horizontal (Fy) components of the GRF were introduced to the computer through an analogue to digital converter (PH-770; DKH, Tokyo) at a sampling frequency of 1 kHz. The details of the analysis of the force-time components during all jumps are shown in Fig 2. Through the measurement of the flight time in the SJ and CMJ, the vertical take-off velocity (Vto_flight time) of the centre of gravity was calculated with the following equation:

Vto_flighttime=1/2⋅flighttime⋅g,

where g is the acceleration of gravity (9.81 m/s^-2^). The jump height (JH) in the SJ and CMJ was derived from Vto_flight time using the following equation:

$$\text{JH}\_\text{flight}\;\text{time}=\text{Vto}\_{\text{flight}\;\text{time}}^{2}\cdot {\left(2\;\text{g}\right)}^{-1}.$$

The minimum vertical GRF (Fz_mini) for the SJ, CMJ and HJ was defined at the point where Fz was the lowest during the contact phase before Fz increased to the maximum vertical GRF (Fz_max). Fz_max for the CMJ and HJ was defined at the point where Fz reached a peak for the first time if two peaks were present. The net vertical impulse (VI) was calculated with the following equation [26,27]:

VI=Fz⋅Δt1−BW⋅Δt1

where Δt1 is the change in time (s) from the starting point where the participant is stationary (start of jump) through the point where the participant takes off and BW is the body weight. Δt1 is also defined as the take-off phase. From VI, the jump height (JH_impulse) in the SJ and CMJ was derived using the following equation [28,29]:

VI=m⋅Vto_impulse

where m and Vto_impulse are the body mass (kg) and the vertical take-off velocity.


$$\text{JH}\_\text{impulse}=\text{Vto}\_{\text{impulse}}^{2}\cdot {\left(2\;\text{g}\right)}^{-1}.$$

The net horizontal impulse (HI) was calculated with the following equation:

HI=Fy⋅Δt1−Fy⋅Δt2

where Δt2 is the change in time (s) from the onset of Fy to the point where the horizontal GRF returned to zero from the negative (posterior) value and became the positive (anterior) value.

Fz_max, Fz_mini, Fy_max, VI and HI were further normalised to the body mass and were represented as rFz_max, rFz_mini, rFy_max, rVI and rHI, respectively.

### The centre of pressure (COP) trajectory

The COP trajectory during the take-off phase of jumping was analysed by applying forces on a force plate. The COP measurements have been described elsewhere [23,30]. The following variables were used to describe the movement of the COP: total length (TL); mean velocity (MV = TL / total time); sway area (SA); maximum range in the antero-posterior direction (AP range); length in antero-posterior direction (AP length); and mean velocity in the antero-posterior direction (AP velocity = AP length / total time). TL and SA were calculated with the following equation [31]:

$$\text{TL}=\sum_{n=1}^{N-1}\sqrt{{\left(AP_{n+1}-AP_{n}\right)}^{2}+{\left(ML_{n+1}-ML_{n}\right)}^{2}}$$

$$\text{SA}=\frac{1}{2}\sum_{n=1}^{N}\left|AP_{n+1}ML_{n}-AP_{n}ML_{n+1}\right|$$

where N is the number of data points during the take-off phase of jumps and n is the COP time series. TL is the total length of the COP trajectory, i.e., the sum of the distance between consecutive points of the COP trajectory. SA was estimated by the area of a convex hull; the sum of the triangulation formed by two points on the COP trajectory was necessary for calculating the convex hull. The AP range was the distance between the anterior and posterior peak displacements.

### Statistical analysis

All data were presented as the means ± SD. The significant differences between the parameters of the two different conditions were tested using the paired Student’s t-test, and the significant differences among the relative differences in 3 jump performance were tested using a one-way repeated-measures analysis of variance with Bonferroni-corrected pairwise post hoc comparisons. The relative GRF values represented changes in the GRF in the take-off phase of the SJ, CMJ and HJ. The effects of FFIM on the relative changes in the GRF in the take-off phase of the SJ, CMJ and HJ were analysed using a 2-way repeated-measures analysis of variance. When an interaction was identified, the Bonferroni-corrected pairwise post hoc comparison was performed. Additionally, a two-sample t-test of statistical parametric mapping (SPM) was used to determine the effects of FFIM on relative values of the GRF in the SJ, CMJ and HJ. SPM analyses were performed using the open-source SPM code (www.spm1d.org) in Matlab (R2018b; MathWorks Inc, Natick, MA, USA) in accordance with a previous study [32]. The effect size (ES: Cohen’s d) was calculated, and ES was evaluated as trivial (0~0.19), small (0.20~0.49), medium (0.50~0.79) and large (0.80 and greater) degrees [33]. The level of statistical significance was set at p < 0.05.

## Results

### Jump performance

**Fig 3:**
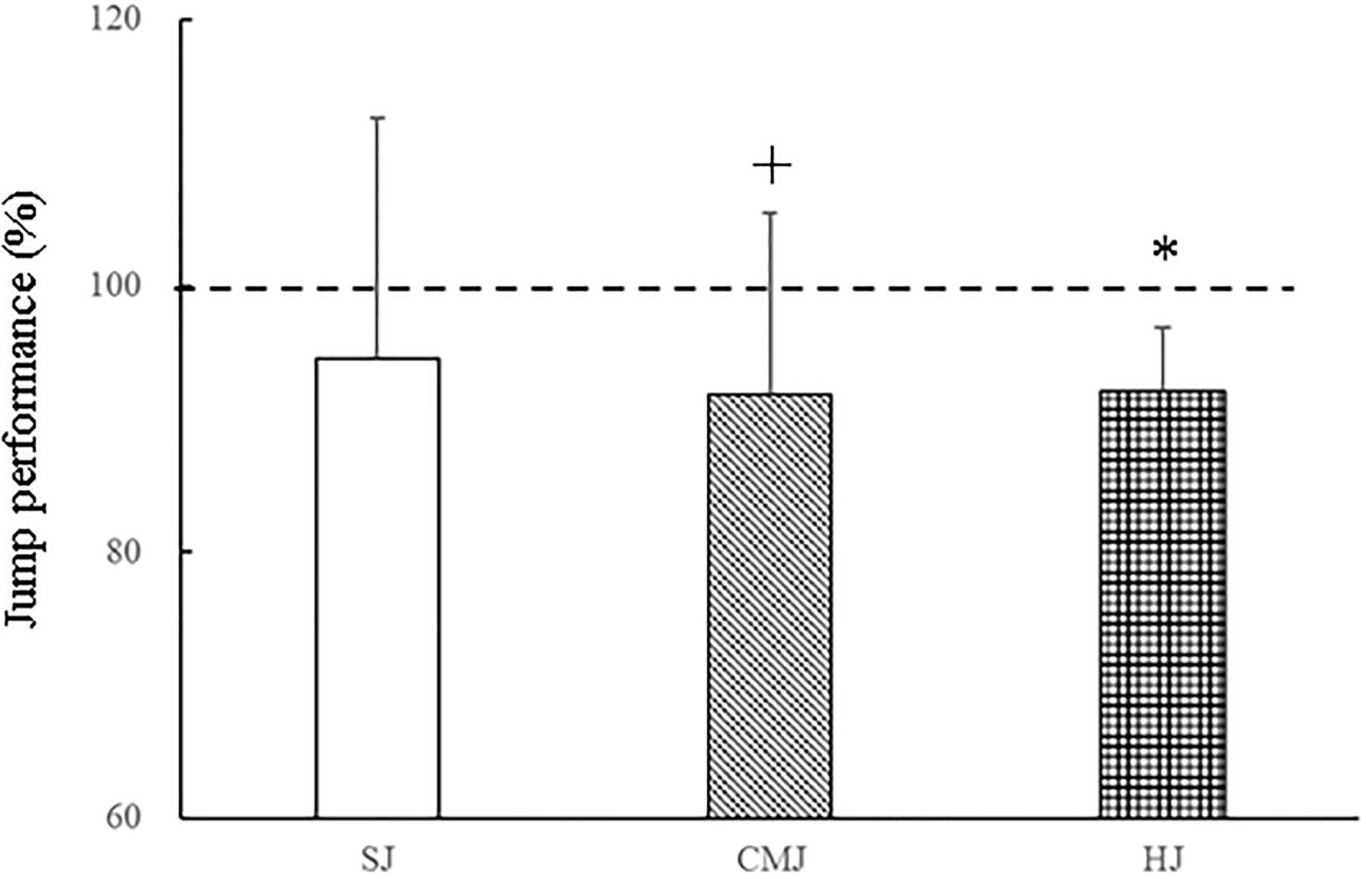
Relative differences in jump performance for the squat jump (SJ), countermovement jump (CMJ) and horizontal jump (HJ) under forefoot immobilisation (FFIM). Values are mean and SD. Jump heights in the SJ and CMJ were calculated from the net vertical impulse (Jump height _impulse). The dashed line at 100% indicates the level without a splint (BARE). + and * denote significant differences between BARE and FFIM at p < 0.05 and p < 0.01, respectively.

Table 1 shows the jump performance of the SJ, CMJ and HJ under the BARE and FFIM conditions. The SJ and CMJ heights were lower under FFIM than under BARE (JH_flight time: p < 0.05 for SJ, p = 0.074 for CMJ; JH_impulse: p = 0.068 for SJ, p < 0.05 for CMJ). JH_flight time was significantly (p < 0.01) higher than JH_impulse in SJ and CMJ under the BARE and FFIM conditions, indicating that the JH_flight time was overestimated [28,29]. The HJ distance under FFIM was significantly shorter than that under BARE (p < 0.01). Fig 3 shows relative differences in jump performance for the SJ, CMJ and HJ under forefoot immobilisation. JH_impulse of FFIM was significantly lower (8.2 ± 13.7%, p < 0.05) than that of BARE for CMJ, whereas for SJ, JH_impulse of FFIM tended to be lower (5.5 ± 18.1%, p = 0.22) than that of BARE. A decrease in HJ distance under FFIM represents 8.0 ± 4.8% (p < 0.01) that of BARE. There were not significant differences in decreased jump performance under FFIM among 3 jump performances.

**Table 1:** Comparison of jump performance during the squat jump (SJ), countermovement jump (CMJ) and horizontal jump (HJ) under barefoot (BARE) and forefoot immobilization (FFIM) conditions.

		BARE	FFIM	P-value	ES
SJ	Jump height_flight time (cm)	17.3 ± 3.9	16.7 ± 3.5	< 0.05	0.559
	Jump height_impulse (cm)	15.5 ± 4.5	14.2 ± 3.7	0.068	0.459
CMJ	Jump height_flight time (cm)	20.5 ± 4.2	19.2 ± 4.1	0.074	0.449
	Jump height_impulse (cm)	18.1 ± 4.6	16.4 ± 3.8	< 0.05	0.556
HJ	Jump distance (cm)	168.9 ± 27.5	155.9 ± 28.4	< 0.01	1.978

Values are mean and SD. ES: Effect size.

### GRF related variables of jumping

**Fig 4:**
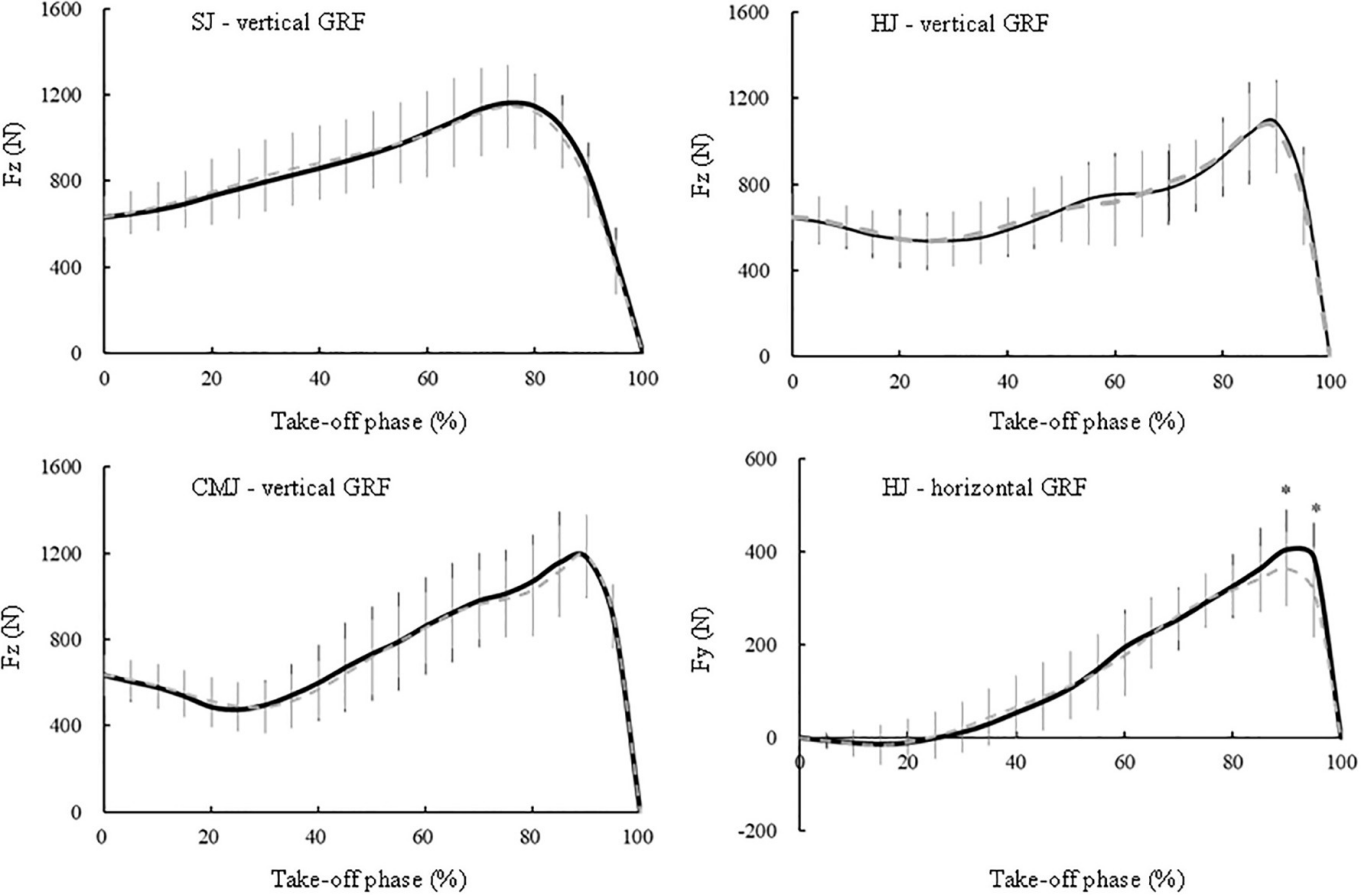
Relative changes in the ground reaction force (GRF) during the take-off phase of the squat jump (SJ), countermovement jump (CMJ) and horizontal jump (HJ) under barefoot (BARE) and forefoot immobilisation (FFIM) conditions. BARE: Black line (mean) and bar (SD); FFIM: Grey dashed line (mean) and grey dotted bar (SD). * denotes a significant difference between BARE and FFIM at p < 0.01.

**Fig 5:**
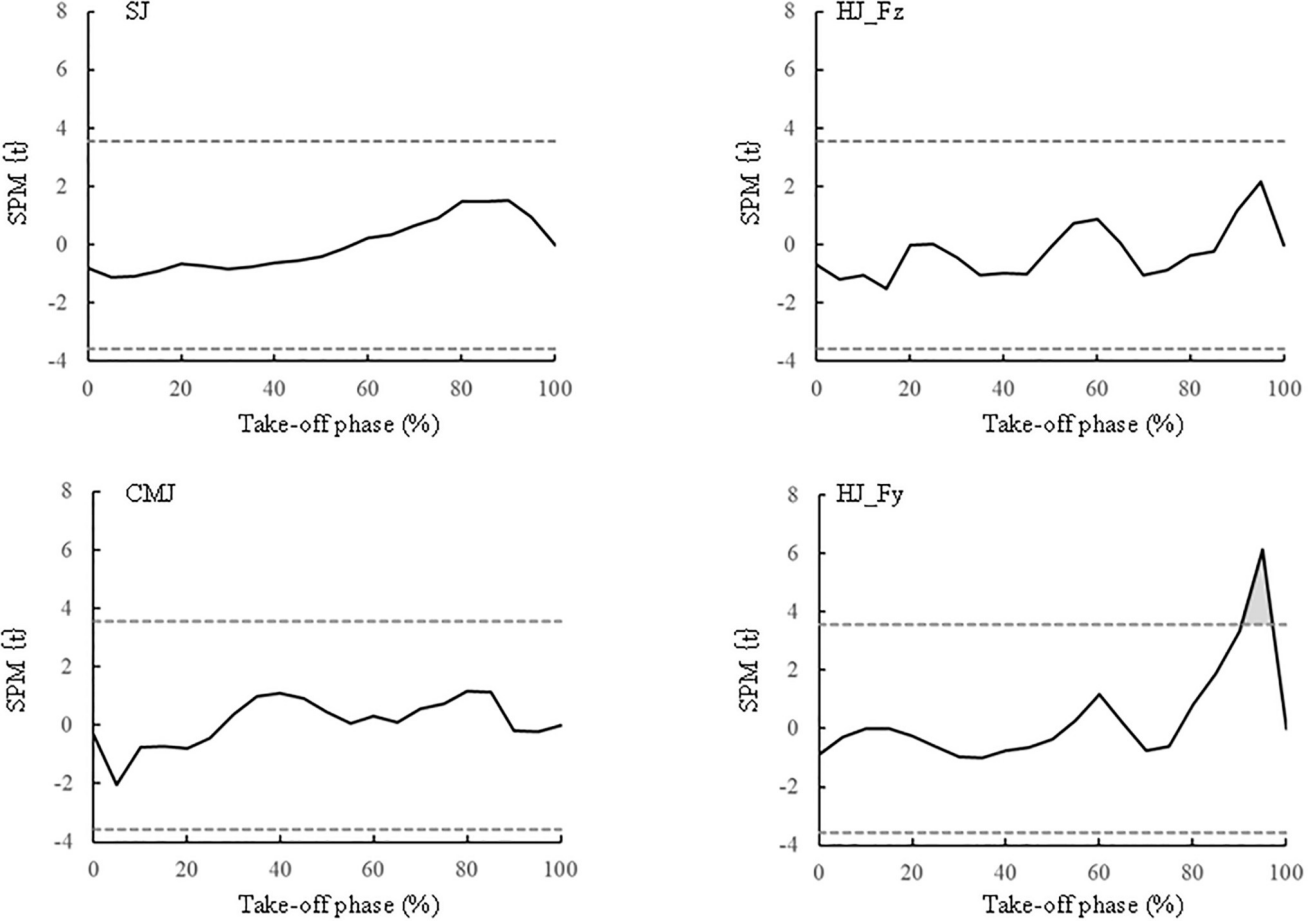
The statistical parametric mapping (SPM) in relative changes in the ground reaction force (GRF) during the take-off phase of the squat jump (SJ), countermovement jump (CMJ) and horizontal jump (HJ). Grey dotted lines are the critical threshold points where are considered significantly different between BARE and FFIM. Grey area is significant difference between BARE and FFIM.

Table 2 summarises the GRF related variables of the SJ, CMJ and HJ under the BARE and FFIM conditions. The relative net vertical impulse under FFIM was lower than that under BARE during the SJ (p = 0.086) and CMJ (p < 0.05). During the HJ, all horizontal GRF variables under FFIM were significantly lower than those under BARE (p < 0.01), whereas none of the vertical GRF variables of the two conditions differed. Fig 4 showed that the relative vertical GRF of the two conditions did not differ significantly at any point of the take-off phase for any of the jumps (p > 0.05); however, the relative horizontal GRF in 90 and 95% of the take-off phase in the HJ under FFIM was significantly lower than that under BARE (p < 0.01). As shown in Fig 5, SPM confirmed that there were no significant differences between BARE and FFIM in the relative vertical GRF in the take-off phase of the SJ, CMJ and HJ (p > 0.05); however, there was significant differences between BARE and FFIM in the relative horizontal GRF in the take-off phase (95%) of the HJ (t > 3.57, p < 0.01).

**Table 2:** Comparison of ground reaction force (GRF) related variables during the take-off phase of the squat jump (SJ), countermovement jump (CMJ) and horizontal jump (HJ) under barefoot (BARE) and forefoot immobilization (FFIM) conditions.

		BARE	FFIM	95% CI	ES
SJ	Maximum vertical GRF (N)	1183.4 ± 172.3	1169.1 ± 201.3	-21.1–49.7	0.201
	Relative maximum vertical GRF (N/kg)	18.4 ± 1.9	18.1 ± 2.1	-0.27–0.83	0.246
	Vertical impulse (N•s)	112.2 ± 14.9	108.3 ± 14.2	-1.37–9.16	0.368
	Relative vertical impulse (N/kg•s)	1.8 ± 0.3	1.7 ± 0.2	-0.01–0.15	0.429
CMJ	Maximum vertical GRF (N)	1155.7 ± 269.3	1116.2 ± 236.8	-42.5–121.4	0.240
	Relative maximum vertical GRF (N/kg)	17.9 ± 3.4	17.3 ± 3.2	-0.66–1.83	0.233
	Minimum vertical GRF (N)	416.2 ± 94.0	407.9 ± 113.0	-32.0–48.7	0.103
	Relative minimum vertical GRF (N/kg)	6.5 ± 1.5	6.3 ± 1.6	-0.39–0.79	0.167
	Vertical impulse (N•s)	121.8 ± 15.0	116.0 ± 13.5 +	0.86–10.8	0.584
	Relative vertical impulse (N/kg•s)	1.9 ± 0.2	1.8 ± 0.2 +	0.01–0.17	0.569
HJ	Maximum vertical GRF (N)	1096.9 ± 214.2	1107.9 ± 182.7	-78.4–56.4	0.081
	Relative maximum vertical GRF (N/kg)	16.9 ± 1.9	17.2 ± 2.1	-1.26–0.74	0.131
	Vertical impulse (N•s)	70.8 ± 16.3	67.8 ± 21.8	-3.68–9.58	0.221
	Relative vertical impulse (N/kg•s)	1.1 ± 0.2	1.1 ± 0.3	-0.05–0.15	0.246
	Maximum horizontal GRF (N)	421.5 ± 93.5	383.1 ± 85.1 *	17.9–58.8	0.932
	Relative maximum horizontal GRF (N/kg)	6.6 ± 1.4	6.0 ± 1.3 *	0.28–0.88	0.954
	Horizontal impulse (N•s)	162.4 ± 27.1	148.8 ± 29.4 *	9.04–18.2	0.982
	Relative horizontal impulse (N/kg•s)	2.5 ± 0.4	2.3 ± 0.4 *	0.14–0.28	1.254

Values are mean and SD. ES: Effect size.

^+^ and * denote significant differences between BARE and FFIM at p < 0.05 and p < 0.01, respectively.

### COP related variables of jumping

Table 3 summarises the COP related variables in the take-off phase of the SJ, CMJ and HJ under the BARE and FFIM conditions. The COP range in the antero-posterior direction under FFIM was significantly larger than that under BARE during the SJ (p < 0.05), whereas it was smaller than that under BARE during the HJ (p < 0.05). Also, the COP range in the anterior direction under FFIM was larger than that under BARE during the SJ (p < 0.05) and CMJ (p < 0.05), and the COP length in the posterior direction under FFIM was significantly shorter than that under BARE during the CMJ (p < 0.05).

**Table 3:** Comparison of the centre of pressure (COP) related variables during the take-off phase of the squat jump (SJ), countermovement jump (CMJ) and horizontal jump (HJ) under barefoot (BARE) and forefoot immobilisation (FFIM) conditions.

		BARE	FFIM	95% CI	ES
SJ	Ground contact time (s)	0.53 ± 0.09	0.52 ± 0.10	-0.07–0.08	0.044
	Total length (cm)	24.2 ± 9.9	24.0 ± 9.0	-4.85–5.22	0.018
	Mean velocity (cm/s)	47.4 ± 22.0	47.3 ± 21.6	-13.9–14.2	0.006
	Sway area (cm^2^)	16.1 ± 10.1	20.2 ± 10.6	-9.82–1.62	0.350
	Antero-posterior range (cm)	11.7 ± 3.1	13.8 ± 4.3 +	-3.92–-0.41	0.613
	Anterior range (cm)	10.7 ± 2.8	12.9 ± 4.3 +	-4.06–-0.30	0.586
	Posterior range (cm)	-0.96 ± 0.95	-0.95 ± 0.97	-0.74–0.71	0.009
	Antero-posterior length (cm)	20.2 ± 8.8	20.4 ± 8.6	-5.16–4.74	0.020
	Anterior length (cm)	13.9 ± 4.0	15.5 ± 4.9	-3.81–0.70	0.333
	Posterior length (cm)	-6.3 ± 5.8	-5.0 ± 5.1	-4.80–2.10	0.189
	Antero-posterior velocity (cm/s)	39.7 ± 19.7	40.4 ± 21.0	-14.2–12.9	0.023
CMJ	Ground contact time (s)	1.09 ± 0.20	1.13 ± 0.22	-0.16–0.09	0.140
	Total length (cm)	32.7 ± 6.9	32.1 ± 5.1	-3.08–4.38	0.086
	Mean velocity (cm/s)	30.5 ± 6.4	29.5 ± 7.9	-3.94–6.08	0.105
	Sway area (cm^2^)	23.2 ± 13.5	19.3 ± 8.2	-2.04–9.78	0.318
	Antero-posterior range (cm)	14.5 ± 2.5	16.6 ± 4.9	-4.91–0.67	0.375
	Anterior range (cm)	11.8 ± 2.7	15.2 ± 5.1 +	-5.85–-0.77	0.639
	Posterior range (cm)	-2.7 ± 2.9	-1.5 ± 1.7	-2.44–0.06	0.472
	Antero-posterior length (cm)	27.4 ± 5.8	27.1 ± 4.3	-3.61–4.07	0.029
	Anterior length (cm)	17.8 ± 3.0	20.5 ± 4.8	-5.46–0.07	0.477
	Posterior length (cm)	-9.5 ± 5.4	-6.6 ± 2.3 +	-5.36–-0.49	0.587
	Antero-posterior velocity (cm/s)	25.7 ± 6.1	25.2 ± 7.6	-4.20–5.23	0.054
HJ	Ground contact time (s)	1.19 ± 0.26	1.12 ± 0.25	-0.06–0.19	0.275
	Total length (cm)	39.9 ± 8.2	38.1 ± 5.4	-2.85–6.46	0.239
	Mean velocity (cm/s)	34.2 ± 6.3	34.7 ± 6.1	-4.99–3.90	0.059
	Sway area (cm^2^)	32.5 ± 13.9	29.2 ± 17.6	-6.67–13.4	0.163
	Antero-posterior range (cm)	22.3 ± 4.5	19.8 ± 3.6 +	0.11–4.76	0.508
	Anterior range (cm)	16.0 ± 4.7	14.4 ± 4.9	-1.04–4.23	0.303
	Posterior range (cm)	-6.3 ± 4.0	-5.4 ± 2.2	-2.96–1.29	0.204
	Antero-posterior length (cm)	35.5 ± 8.1	33.9 ± 4.8	-2.74–5.93	0.216
	Anterior length (cm)	25.3 ± 4.6	23.5 ± 4.0	-0.58–4.12	0.405
	Posterior length (cm)	-10.2 ± 5.1	-10.4 ± 4.0	-2.81–3.15	0.033
	Antero-posterior velocity (cm/s)	30.8 ± 5.9	30.9 ± 5.7	-4.36–3.96	0.023

Values are presented as the mean and SD. ES: Effect size.

^+^ denotes a significant difference between BARE and FFIM at p < 0.05.

## Discussion

The present study revealed for the first time the mechanical role of MTP joint during vertical and horizontal jump performance. The novel findings from this study were that the vertical and horizontal jump performance decreased under the forefoot immobilisation condition, indicating the mechanical contribution of MTP joint motion during jumping was presented. During the HJ, all the horizontal GRF variables and the relative horizontal GRF in the final take-off phase decreased significantly under forefoot immobilisation, although none of the vertical GRF variables was affected. Additionally, under forefoot immobilisation, the COP range in the antero-posterior direction in the take-off phase of the HJ decreased, whereas its range in the anterior direction for the SJ and CMJ increased. The results of this study indicate that MTP joint motion can play an important role in regulating force-generating capacities of toe flexor muscles in the take-off phase of human jumping, especially in the horizontal direction of horizontal jumping.

The vertical and horizontal jump performance decreased under forefoot immobilisation, which could be due to the reduction of force generation of toe flexor muscles at the MTP joints in the take-off phase of jumping. The different contributions of the toe flexor muscles during the vertical and horizontal jumps have been discussed previously [5]. The results of this study showed that the horizontal jump performance required force generation of toe flexor muscles at the MTP joints to a large extent. The MTP moment and dorsiflexion in the HJ are significantly larger than those in the VJ, indicating that toe flexor muscles contribute force to movements in leaned-forward positions [5,34]. Moreover, jump performance in the countermovement action of the CMJ and HJ was significantly impaired by forefoot immobilization (Fig 3). A vertical force during the countermovement action stretches the toe flexor muscle-tendon complex under the truss structure of the foot, which in turn increases the force generation in locomotion [35]. Force production of the muscle at the beginning of the movement can enhance when muscles are pre-stretched [36]. When activated muscle fibres are forcefully stretched, muscles can produce a higher force than no stretching condition [37]. The mechanical factor of muscle stretching has the propulsive effect at the beginning of the muscle shortening. When the foot arch is compressed by the load, the plantar muscle-tendon complex is stretched and this interaction amplifies the force-generating capacity of the toe flexors [15]. Therefore, it is conceivable that MTP joint motion with the foot arch dynamics is important for force generation of toe flexor muscles in the final take-off phase of jumping.

**Fig 6:**
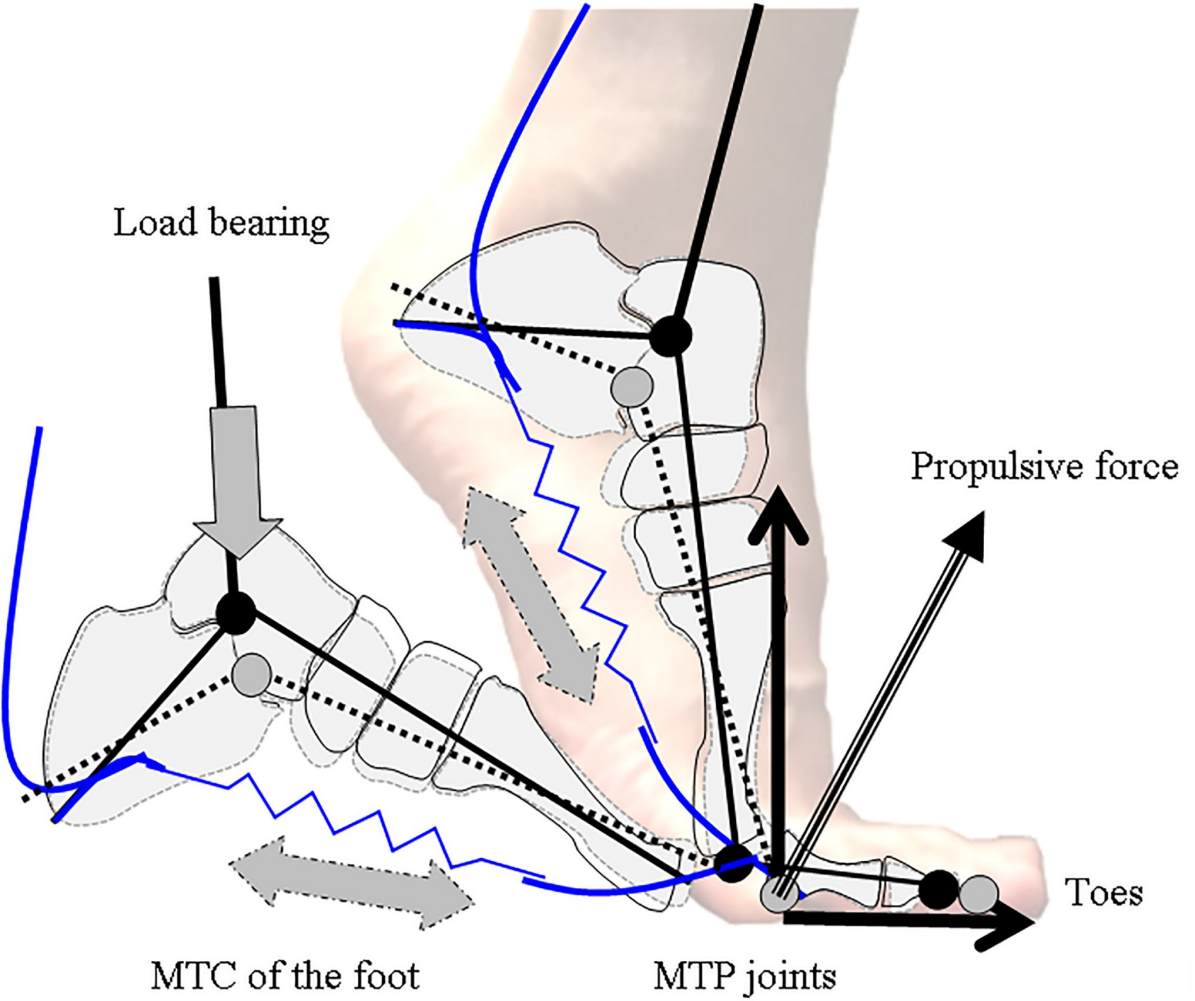
Schematic model of the force amplification mechanism of the toe flexors. MTC, Muscles, tendon, fascia & aponeurosis complex; MTP, metatarsophalangeal joints. Gray arrow and dashed lines show the schema in which the vertical load applied to the talus bone is transmitted to the metatarsal and the calcaneus bones, thenceforward increasing in the tension of the MTC of the toe flexors and enhancing the force generation at the push-off.

Incidentally, why is the impact of MTP motion most prominent for the HJ? The MTP joints motion was significant for generating the forces on the ground in the final take-off phase of the HJ (Figs 4 & 5). During the take-off phase of the HJ, the ability to develop the horizontal force on the ground was required for the final acceleration of the body in the forward direction [5,34,38]. Jump performance is enhanced by multi-segment force transmission of all leg joints, which rapidly generates a large force on the ground [39]. Because the human foot is the terminal of a closed kinetic chain in the lower limb, it integrates the forces from all the joints of the leg in the take-off phase of jumping. During the final take-off phase of jumping, the heel is lifted from the ground, and the hallux and lesser toes are passively hyper-extended or buckled at the MTP joints. This windlass mechanism induces the effect of toe dorsiflexion on plantar aponeurosis tensile strain [40]. The MTP joints dorsiflexion could maintain the pre-stretched toe flexor muscle and tendon complex under the foot arch [3,4]. The toe flexor muscles at the MTP joints help to support the foot arches and contribute to the generation of force at the MTP joints [41]. When the toes are hyper-extended at the MTP joints, the stretched and stiffed toe flexor muscle and tendon complex is able to potentiate propulsive force transmission [42]. The foot arch stiffness improves propulsive force via reutilising the elastic energy stored in the plantar muscles and tendon complex during running [43]. In addition to this, when the MTP joints dorsiflex during running and sprinting, they absorb a significant amount of energy [44]. Therefore, the toes could explosively push off the ground during horizontal jumping if force was integrated and generated at MTP joints. Overall, the force generated at the MTP joints could be released from the forefoot in the forward direction during the final take-off phase of horizontal jumping when MTP joints immediately snaps-backs (Fig 6).

A characteristic COP trajectory in the take-off phase of jumping was altered by foot immobilisation. Intriguingly, under foot immobilisation, the COP in the anterio-posterior direction in the SJ and CMJ shifted into the anterior axis direction, whereas the COP in the antero-posterior direction in the HJ shifted in to the central axis direction. The centre of force application shifts in the anterior direction when the body moves forward in horizontal jumping [5]. Similarly, the COP trajectory in running moves forward the forefoot crossing the MTP joint [18,45]. Accordingly, foot immobilisation could cause a less leaned-forward position and decrease the forward propulsion force on the ground in the push off phase of the HJ. Contrary, it is interesting to know that the COP range in the anterior direction in the SJ and CMJ increased under forefoot immobilisation. This could be an increase in force generation from the large extensor muscles of the lower limb during these jumps in order to maintain their jump performance. It has been shown that alternating the bi-articular muscle activities of the lower limb is observed in different jump directions [46]: different bi-articular muscle activities influence the force production of the large muscles of the leg in the take-off phase of jumping. The bi-articular muscles of the leg could help transfer energy from one segment to another [47]. This transfer of the external force of the leg to the foot can be assisted by the bi-articular muscles of the foot. These suggest that forefoot immobilisation may alternate the muscle coordination pattern of the lower limb and the force application on the ground in the foot.

There were several inherent limitations in this study. Without kinematic and electromyography assessments of the lower limb and foot, it is difficult to determine whether forefoot immobilisation affects MTP joints motion alone during jumping because all forefoot joints motion was immobilised. Although toe flexor muscles generate force at the MTP and interphalangeal joints of the forefoot [48], many intrinsic toe flexor muscles are involved at the MTP joint. Over the years, these muscles have adapted from gripping function to generating a propulsion force with respect to the ground during bipedal locomotion [49]. Future research should clarify the relative contribution of intrinsic and extrinsic muscles during force generation at the MTP and interphalangeal joints of the forefoot during jumping. Besides, the forefoot immobilisation could alter the anatomical linkage between muscles at the ankle and all forefoot joints and the length-tension relation of their muscle fibres. This might cause to reduce the ability to develop the force generation of the toe flexor and the plantar flexor muscles in the take-off phase of jumping. In fact, because ankle joint angles influences the length-tension relation of toe flexor muscles [34,50], limiting the movements of the ankle joint using ankle braces impaired force generation of the toe flexor and plantar flexor muscles [51] and countermovement jump performance [52,53]. Additionally, taping the plantar surface might reduce the plantar cutaneous sensitivity and be associated with a loss of postural stability in the take-off phase of jumping. The sensory system in the foot plays an important role in maintaining postural stability [54]. Although the order of the jumps has no order effect [55,56], it may be considered to randomize the order of the jumps for future studies. Therefore, a prospective controlled study including a systematic method to impair the toe flexor muscles is warranted. Despite these limitations, our findings can provide motivation for advancing our understanding of the biomechanical significance of the specific mechanism of the MTP joint in human jumping.

## Conclusions

The results of this study confirmed the importance of the mechanical contribution of the MTP joints motion during human jumping. MTP joint motion requires the ability to integrate and generate force in the take-off phase of human countermovement jumping, especially in horizontal jumping. For a practical point of view, because jump performance decreased under forefoot immobilisation, more attention should be paid to how the mechanical role of the MTP joint affects the wearing of shoes and exercise training for athletes. Wearing a shoe may cause the foot not to push off efficiently from the ground during biped locomotion. We should rethink how the human foot evolved for bipedal upright locomotion without wearing shoes.

## Abbreviations

COP: centre of pressure
CMJ: countermovement jump
FFIM: forefoot immobilisation
GRF: ground reaction force
HJ: horizontal jump
MTP: metatarsophalangeal
SJ: squat jump
VJ: vertical jump
